# 1,8-Bis(4-amino­benzo­yl)-2,7-dimeth­oxy­naphthalene

**DOI:** 10.1107/S1600536810041346

**Published:** 2010-10-23

**Authors:** Takahiro Nishijima, Kotaro Kataoka, Atsushi Nagasawa, Akiko Okamoto, Noriyuki Yonezawa

**Affiliations:** a Department of Organic and Polymer Materials Chemistry, Tokyo University of Agriculture & Technology, 2-24-16 Naka-machi, Koganei, Tokyo 184-8588, Japan

## Abstract

The title compound {systematic name: [8-(4-aminobenzoyl)-2,7-dimethoxynaphthalen-1-yl](4-aminophenyl)methanone}, C_26_H_22_O_4_N_2_, possesses crystallographically imposed twofold symmetry, with two C atoms lying on the rotation axis. In the crystal, the mol­ecules inter­act through inter­molecular N—H⋯O hydrogen bonds between the amino and meth­oxy groups on the naphthalene ring systems and N—H⋯π inter­actions between the amino groups and the naphthalene rings. Furthermore, weak C—H⋯O hydrogen bonds and π–π stacking inter­actions between the benzene rings are observed. The centroid–centroid and inter­planar distances between the benzene rings of the aroyl group and the naphthalene ring systems of adjacent mol­ecules are 3.6954 (8) and 3.2375 (5) Å, respectively. The dihedral angle between the mean planes of the benzene ring and the naphthalene ring system is 83.59 (5)°. The benzene ring and the carbonyl group in the benzoyl unit are almost coplanar [C—C—C—O torsion angle = 175.91 (10)°].

## Related literature

For the formation reaction of aroylated naphthalene compounds *via* electrophilic aromatic aroylation of 2,7-dimeth­oxy­naphthalene, see: Okamoto & Yonezawa (2009 ▶). For related structures, see: Muto *et al.* (2010 ▶); Nakaema *et al.* (2007 ▶, 2008 ▶); Watanabe *et al.* (2010*a* ▶,*b* ▶). For work-up procedure in the preparation of the title compound, see: Bellamy *et al.* (1984 ▶).
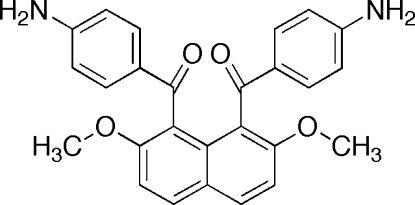


## Experimental

### 

#### Crystal data

C_26_H_22_N_2_O_4_
*M* *_r_* = 426.46
Monoclinic, 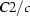

*a* = 14.2996 (3) Å
*b* = 10.2811 (2) Å
*c* = 15.4306 (3) Å
β = 114.523 (1)°
*V* = 2063.90 (7) Å^3^
*Z* = 4
Cu *K*α radiation
μ = 0.76 mm^−1^
*T* = 193 K
0.40 × 0.30 × 0.10 mm

#### Data collection

Rigaki R-AXIS RAPID diffractometer
Absorption correction: numerical (*NUMABS*; Higashi, 1999 ▶) *T*_min_ = 0.751, *T*_max_ = 0.928
17453 measured reflections
1892 independent reflections
1690 reflections with *I* > 2σ(*I*)
*R*_int_ = 0.056

#### Refinement

*R*[*F* ^2^ > 2σ(*F* ^2^)] = 0.043
*wR*(*F* ^2^) = 0.114
*S* = 1.12
1892 reflections
178 parameters
H atoms treated by a mixture of independent and constrained refinement
Δρ_max_ = 0.24 e Å^−3^
Δρ_min_ = −0.37 e Å^−3^

### 

Data collection: *PROCESS-AUTO* (Rigaku, 1998 ▶); cell refinement: *PROCESS-AUTO*; data reduction: *CrystalStructure* (Rigaku/MSC, 2004 ▶); program(s) used to solve structure: *SIR2004* (Burla *et al.*, 2005 ▶); program(s) used to refine structure: *SHELXL97* (Sheldrick, 2008 ▶); molecular graphics: *ORTEPIII* (Burnett & Johnson, 1996 ▶); software used to prepare material for publication: *SHELXL97*.

## Supplementary Material

Crystal structure: contains datablocks I, New_Global_Publ_Block. DOI: 10.1107/S1600536810041346/rz2496sup1.cif

Structure factors: contains datablocks I. DOI: 10.1107/S1600536810041346/rz2496Isup2.hkl

Additional supplementary materials:  crystallographic information; 3D view; checkCIF report

## Figures and Tables

**Table 1:** Hydrogen-bond geometry (Å, °) *Cg* is the centroid of the C8–C13 ring.

*D*—H⋯*A*	*D*—H	H⋯*A*	*D*⋯*A*	*D*—H⋯*A*
N1—H4⋯O1^i^	0.93 (2)	2.26 (2)	3.1708 (17)	169.1 (18)
C14—H14*B*⋯O2^ii^	0.96	2.57	3.5013 (18)	165
N1—H3⋯*Cg*^iii^	0.92 (2)	2.50 (2)	3.3301 (13)	149.8 (18)

Symmetry codes: (i) 
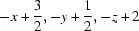
; (ii) 
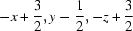
; (iii) 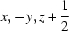.

**Fig. 1.**
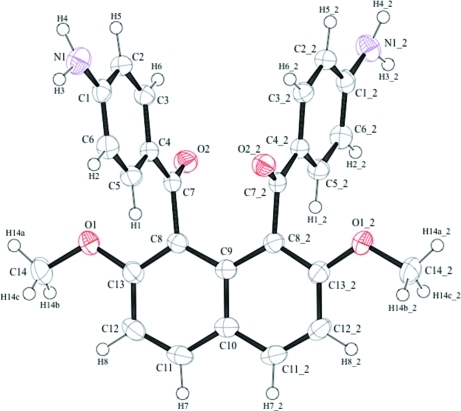
Molecular structure of title compound with displacement ellipsoids drawn at the 50% probability level. The symbol_2 refers to symmetry code: -x + 1, y, -z + 3/2.

**Fig. 2.**
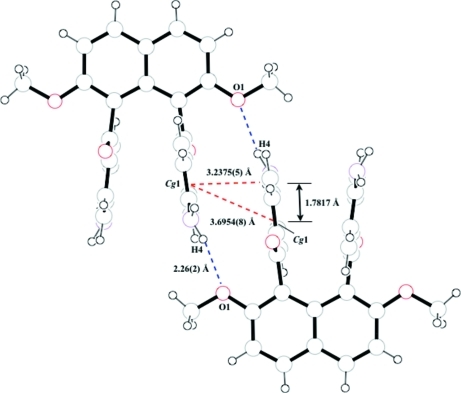
Side view of the N—H···O hydrogen bond (blue dashed lines) and the π—π interaction (red dashed lines).

**Fig. 3.**
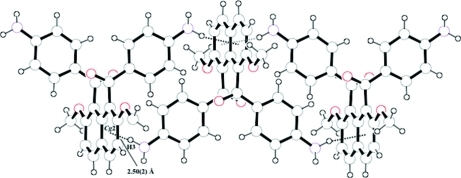
Side view of the N—H···π interactions (dashed lines).

**Fig. 4.**
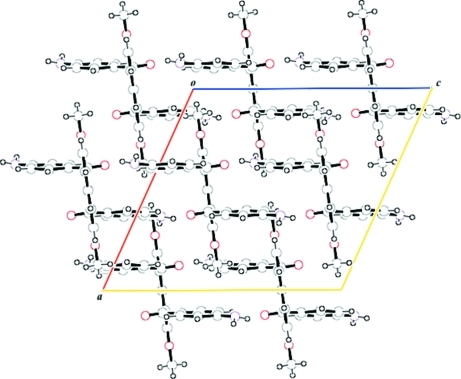
A partial packing diagram of the title compound, viewed down the b axis.

## supplementary crystallographic information

### Comment

In the course of our study on selective electrophilic aromatic aroylation of naphthalene core, *peri*-aroylnaphthalene compounds have proved to be formed regioselectively by the aid of suitable acidic mediators (Okamoto & Yonezawa, 2009). The aroyl groups at 1,8-positions of the naphthalene rings in these compounds are oriented in opposite direction. The aromatic rings in this molecule are clarified to be assembled with non-coplanar configuration resulting in partial disruption of π-conjugated ring systems. Recently, we reported the X-ray crystal structures of 1,8-diaroylated 2,7-dimethoxynaphthalenes such as 1,8-bis(4-chlorobenzoyl)-2,7-dimethoxynaphthalene (Nakaema *et al.*, 2007), 1,8-dibenzoyl-2,7-dimethoxynaphthalene (Nakaema *et al.*, 2008), bis(4-bromophenyl) (2,7-dimethoxynaphthalene-1,8-diyl)dimethanone (Watanabe *et al.*, 2010*a*), (2,7-dimethoxynaphthalene-1,8-diyl)bis(4-fluorophenyl)dimethanone (Watanabe *et al.*, 2010*b*) and 1,8-bis(4-methylbenzoyl)-2,7-dimethoxynaphthalene (Muto *et al.*, in press). As a part of our continuous study on the molecular structures of this kind of homologous molecules, the X-ray crystal structure of title compound, (I), a *peri*-aroylnaphthalene bearing amino groups, is discussed in this article.

An *ORTEPIII* (Burnett & Johnson, 1996) plot of title compound is displayed in Fig. 1. The molecule of (I) lies across a crystallographic 2-fold axis so that the asymmetric unit contains one-half of the molecule. Thus, the two benzoyl groups are situated in *anti* orientation. The benzoyl groups are twisted away from the naphthalene moiety, and the dihedral angle is 83.59 (5)°. The torsion angle between the carbonyl group and the naphthalene ring is -89.52 (13)° [O2—C7—-C8—-C13], and that between the carbonyl group and the benzene ring is 175.91 (10)° [C5—C4—C7—O2].

The molecular packing of (I) is mainly stabilized by intermolecular hydrogen bond and van der Waals interaction (Table 1).The amino groups interact with the methoxy groups [N1—H4···O1^i^ = 2.26 (2) Å; symmetry code: (i)-*x* + 3/2, -*y* - 1/2, -*z* + 2] of the adjacent molecules along the *b* axis (Fig. 2). Moreover, molecules are linked by N—H···π interactions along the *c* axis (Fig. 3). Besides, relatively weak C—H···O hydrogen bonding, C14—H14B···O2^ii^[symmetry code: (ii) -x + 3/2, *y* - 1/2, -*z* + 3/2], and a π—π stacking interaction are observed. In the packing, the molecules form the column structure of stacked naphthalene rings by π—π interaction perpendicular to the *bc* plane with alignment by N—H···π interaction and C—H···O hydrogen bonding along the *c* axis of the unit cell (Fig. 4).

### Experimental

The title compound was prepared by reduction reaction of 1,8-bis(4-nitrobenzoyl)-2,7-dimethoxynaphthalene (486.4 mg, 1.0 mmol), which was obtained *via* electrophilic aromatic diaroylation reaction of 2,7-dimethoxynaphthalene with 4-nitrobenzoyl chloride, with stannous chloride dihydrate (2.256 g, 10 mmol) in EtOH (8.0 ml) at 343 K for 2 h. The reaction mixture was worked up by reference to the previously outlined procedure (Bellamy *et al.*, 1984). Isolated yield 96%. Brown single crystals suitable for X-ray diffraction were obtained by recrystallization from methanol.

Spectral data:^1^H NMR (300 MHz, CDCl_3_) δ 3.71 (6*H*, s), 4.02 (4*H*, s), 6.54 (4*H*, broad), 7.18 (2*H*, d, *J* = 8.7 Hz), 7.53 (4*H*, broad), 7.88 (2*H*, d, *J* = 9.6 Hz); ^13^C NMR (75 MHz, DMSO) δ 56.37, 111.59. 112.22, 122.11, 125.06. 127.41, 128.86, 131.15, 131.29, 153.09, 155.06, 192.49; IR (KBr): 3463.53 (N—H), 3374.82 (N—H), 1644.98 (C═O), 1595.81 (Ar), 1508.06 (Ar)cm^-1^; HRMS (m/*z*): [*M* + H]^+^ Calcd for C_26_H_23_O_4_N_2_, 427.1658; found, 427.1633; m.p.= 580.5–583.0 K(decomp).

### Refinement

All the H-atoms could be located in difference Fourier maps. The amine and aromatic hydrogen atoms were freely refined. The hydrogen atom of methyl groups were subsequently refined as riding atoms with C—H = 0.96 Å and with *U*_iso_(H) = 1.2*U*_eq_(C).

### Crystal data

**Table:**

C_26_H_22_N_2_O_4_	*F*(000) = 896
*M**_r_* = 426.46	*D*_x_ = 1.372 Mg m^−^^3^
Monoclinic, *C*2/*c*	Melting point = 580.5–583.0 K
Hall symbol: -C 2yc	Cu *K*α radiation, λ = 1.54187 Å
*a* = 14.2996 (3) Å	Cell parameters from 11162 reflections
*b* = 10.2811 (2) Å	θ = 3.1–68.2°
*c* = 15.4306 (3) Å	µ = 0.76 mm^−^^1^
β = 114.523 (1)°	*T* = 193 K
*V* = 2063.90 (7) Å^3^	Platelet, brown
*Z* = 4	0.40 × 0.30 × 0.10 mm

### Data collection

**Table:**

Rigaki R-AXIS RAPID diffractometer	1892 independent reflections
Radiation source: rotating anode	1690 reflections with *I* > 2σ(*I*)
graphite	*R*_int_ = 0.056
Detector resolution: 10.00 pixels mm^-1^	θ_max_ = 68.2°, θ_min_ = 5.5°
ω scans	*h* = −17→17
Absorption correction: numerical (*NUMABS*; Higashi, 1999)	*k* = −12→12
*T*_min_ = 0.751, *T*_max_ = 0.928	*l* = −18→18
17453 measured reflections	

### Refinement

**Table:**

Refinement on *F*^2^	Primary atom site location: structure-invariant direct methods
Least-squares matrix: full	Secondary atom site location: difference Fourier map
*R*[*F*^2^ > 2σ(*F*^2^)] = 0.043	Hydrogen site location: inferred from neighbouring sites
*wR*(*F*^2^) = 0.114	H atoms treated by a mixture of independent and constrained refinement
*S* = 1.12	*w* = 1/[σ^2^(*F*_o_^2^) + (0.0679*P*)^2^ + 0.6125*P*] where *P* = (*F*_o_^2^ + 2*F*_c_^2^)/3
1892 reflections	(Δ/σ)_max_ = 0.001
178 parameters	Δρ_max_ = 0.24 e Å^−^^3^
0 restraints	Δρ_min_ = −0.37 e Å^−^^3^

### Special details

**Table:**

Geometry. All e.s.d.'s (except the e.s.d. in the dihedral angle between two l.s. planes) are estimated using the full covariance matrix. The cell e.s.d.'s are taken into account individually in the estimation of e.s.d.'s in distances, angles and torsion angles; correlations between e.s.d.'s in cell parameters are only used when they are defined by crystal symmetry. An approximate (isotropic) treatment of cell e.s.d.'s is used for estimating e.s.d.'s involving l.s. planes.
Refinement. Refinement of *F*^2^ against ALL reflections. The weighted *R*-factor *wR* and goodness of fit *S* are based on *F*^2^, conventional *R*-factors *R* are based on *F*, with *F* set to zero for negative *F*^2^. The threshold expression of *F*^2^ > σ(*F*^2^) is used only for calculating *R*-factors(gt) *etc*. and is not relevant to the choice of reflections for refinement. *R*-factors based on *F*^2^ are statistically about twice as large as those based on *F*, and *R*- factors based on ALL data will be even larger.

### Fractional atomic coordinates and isotropic or equivalent isotropic displacement parameters (Å^2^)

**Table:**

	*x*	*y*	*z*	*U*_iso_*/*U*_eq_	
O1	0.77436 (7)	−0.11857 (9)	0.88398 (7)	0.0426 (3)	
O2	0.62425 (7)	0.07379 (8)	0.72735 (6)	0.0341 (3)	
N1	0.63099 (9)	0.33681 (13)	1.10437 (8)	0.0405 (3)	
C1	0.62489 (9)	0.26028 (13)	1.02908 (8)	0.0307 (3)	
C2	0.63071 (9)	0.31736 (12)	0.94876 (9)	0.0311 (3)	
C3	0.62524 (9)	0.24184 (12)	0.87347 (8)	0.0293 (3)	
C4	0.61301 (8)	0.10665 (11)	0.87440 (8)	0.0268 (3)	
C5	0.60412 (9)	0.05115 (13)	0.95329 (9)	0.0311 (3)	
C6	0.60986 (9)	0.12582 (13)	1.02970 (9)	0.0333 (3)	
C7	0.61101 (8)	0.02784 (12)	0.79446 (8)	0.0266 (3)	
C8	0.59675 (9)	−0.11834 (12)	0.79669 (8)	0.0278 (3)	
C9	0.5000	−0.18296 (16)	0.7500	0.0267 (4)	
C10	0.5000	−0.32223 (16)	0.7500	0.0302 (4)	
C11	0.59352 (11)	−0.39011 (13)	0.79714 (9)	0.0355 (3)	
C12	0.68471 (11)	−0.32756 (13)	0.84270 (9)	0.0366 (3)	
C13	0.68596 (9)	−0.19016 (13)	0.84184 (9)	0.0328 (3)	
C14	0.86976 (11)	−0.18638 (16)	0.93136 (11)	0.0495 (4)	
H14A	0.9248	−0.1246	0.9574	0.059*	
H14B	0.8815	−0.2415	0.8866	0.059*	
H14C	0.8669	−0.2385	0.9818	0.059*	
H1	0.5936 (11)	−0.0400 (16)	0.9542 (10)	0.038 (4)*	
H2	0.6079 (12)	0.0862 (14)	1.0838 (11)	0.039 (4)*	
H3	0.6425 (15)	0.2952 (19)	1.1606 (14)	0.068 (6)*	
H4	0.6672 (15)	0.413 (2)	1.1123 (13)	0.065 (5)*	
H5	0.6425 (11)	0.4089 (16)	0.9514 (10)	0.038 (4)*	
H6	0.6305 (11)	0.2772 (14)	0.8178 (10)	0.034 (3)*	
H7	0.5900 (12)	−0.4824 (17)	0.7944 (11)	0.047 (4)*	
H8	0.7479 (13)	−0.3756 (16)	0.8739 (11)	0.048 (4)*	

### Atomic displacement parameters (Å^2^)

**Table:**

	*U*^11^	*U*^22^	*U*^33^	*U*^12^	*U*^13^	*U*^23^
O1	0.0267 (5)	0.0363 (6)	0.0529 (6)	0.0065 (4)	0.0048 (4)	0.0005 (4)
O2	0.0404 (5)	0.0317 (5)	0.0299 (5)	−0.0019 (4)	0.0141 (4)	0.0015 (4)
N1	0.0409 (7)	0.0464 (7)	0.0354 (6)	−0.0038 (6)	0.0170 (5)	−0.0089 (5)
C1	0.0217 (6)	0.0375 (7)	0.0311 (6)	0.0004 (5)	0.0091 (5)	−0.0033 (5)
C2	0.0309 (6)	0.0258 (7)	0.0348 (7)	−0.0006 (5)	0.0117 (5)	−0.0005 (5)
C3	0.0289 (6)	0.0275 (7)	0.0298 (6)	0.0001 (5)	0.0106 (5)	0.0034 (5)
C4	0.0221 (6)	0.0258 (6)	0.0291 (6)	0.0002 (4)	0.0074 (4)	0.0010 (4)
C5	0.0296 (6)	0.0279 (7)	0.0350 (7)	−0.0015 (5)	0.0126 (5)	0.0032 (5)
C6	0.0321 (6)	0.0383 (7)	0.0308 (6)	−0.0010 (5)	0.0144 (5)	0.0039 (5)
C7	0.0198 (5)	0.0271 (6)	0.0281 (6)	0.0002 (4)	0.0053 (4)	0.0016 (4)
C8	0.0305 (6)	0.0259 (7)	0.0271 (6)	0.0023 (5)	0.0120 (5)	0.0006 (4)
C9	0.0329 (9)	0.0249 (9)	0.0241 (8)	0.000	0.0136 (7)	0.000
C10	0.0407 (10)	0.0258 (9)	0.0278 (8)	0.000	0.0180 (7)	0.000
C11	0.0520 (8)	0.0235 (7)	0.0332 (7)	0.0058 (5)	0.0200 (6)	0.0030 (5)
C12	0.0408 (8)	0.0318 (7)	0.0350 (7)	0.0124 (6)	0.0135 (6)	0.0053 (5)
C13	0.0319 (7)	0.0319 (7)	0.0321 (6)	0.0033 (5)	0.0108 (5)	0.0004 (5)
C14	0.0308 (7)	0.0487 (9)	0.0550 (9)	0.0133 (6)	0.0040 (6)	−0.0041 (7)

### Geometric parameters (Å, °)

**Table:**

O1—C13	1.3712 (15)	C6—H2	0.939 (15)
O1—C14	1.4331 (15)	C7—C8	1.5189 (17)
O2—C7	1.2223 (14)	C8—C13	1.3857 (17)
N1—C1	1.3756 (16)	C8—C9	1.4308 (14)
N1—H3	0.92 (2)	C9—C8^i^	1.4308 (14)
N1—H4	0.92 (2)	C9—C10	1.432 (2)
C1—C6	1.3996 (19)	C10—C11^i^	1.4132 (16)
C1—C2	1.4045 (17)	C10—C11	1.4132 (16)
C2—C3	1.3724 (17)	C11—C12	1.359 (2)
C2—H5	0.954 (16)	C11—H7	0.950 (18)
C3—C4	1.4017 (17)	C12—C13	1.4128 (19)
C3—H6	0.964 (14)	C12—H8	0.965 (17)
C4—C5	1.3972 (16)	C14—H14A	0.9600
C4—C7	1.4662 (16)	C14—H14B	0.9600
C5—C6	1.3806 (18)	C14—H14C	0.9600
C5—H1	0.950 (16)		
			
C13—O1—C14	118.41 (11)	C13—C8—C9	120.11 (12)
C1—N1—H3	117.1 (12)	C13—C8—C7	115.75 (10)
C1—N1—H4	115.9 (12)	C9—C8—C7	123.98 (11)
H3—N1—H4	113.7 (17)	C8—C9—C8^i^	124.66 (15)
N1—C1—C6	121.01 (12)	C8—C9—C10	117.67 (8)
N1—C1—C2	120.00 (12)	C8^i^—C9—C10	117.67 (8)
C6—C1—C2	118.97 (11)	C11^i^—C10—C11	120.81 (17)
C3—C2—C1	120.48 (12)	C11^i^—C10—C9	119.59 (8)
C3—C2—H5	122.7 (8)	C11—C10—C9	119.59 (8)
C1—C2—H5	116.7 (8)	C12—C11—C10	122.17 (13)
C2—C3—C4	121.05 (11)	C12—C11—H7	121.2 (9)
C2—C3—H6	122.9 (9)	C10—C11—H7	116.6 (10)
C4—C3—H6	116.1 (9)	C11—C12—C13	118.78 (12)
C5—C4—C3	118.09 (11)	C11—C12—H8	121.0 (10)
C5—C4—C7	122.03 (11)	C13—C12—H8	120.2 (10)
C3—C4—C7	119.88 (11)	O1—C13—C8	115.30 (11)
C6—C5—C4	121.49 (12)	O1—C13—C12	123.02 (11)
C6—C5—H1	119.3 (9)	C8—C13—C12	121.67 (12)
C4—C5—H1	119.2 (9)	O1—C14—H14A	109.5
C5—C6—C1	119.88 (11)	O1—C14—H14B	109.5
C5—C6—H2	120.3 (9)	H14A—C14—H14B	109.5
C1—C6—H2	119.7 (9)	O1—C14—H14C	109.5
O2—C7—C4	122.92 (11)	H14A—C14—H14C	109.5
O2—C7—C8	118.10 (10)	H14B—C14—H14C	109.5
C4—C7—C8	118.92 (10)		
			
N1—C1—C2—C3	179.66 (11)	C7—C8—C9—C8^i^	−6.37 (8)
C6—C1—C2—C3	−2.20 (18)	C13—C8—C9—C10	−1.54 (11)
C1—C2—C3—C4	0.48 (18)	C7—C8—C9—C10	173.63 (8)
C2—C3—C4—C5	1.46 (17)	C8—C9—C10—C11^i^	−178.56 (8)
C2—C3—C4—C7	−177.87 (10)	C8^i^—C9—C10—C11^i^	1.44 (8)
C3—C4—C5—C6	−1.70 (17)	C8—C9—C10—C11	1.44 (8)
C7—C4—C5—C6	177.61 (10)	C8^i^—C9—C10—C11	−178.56 (8)
C4—C5—C6—C1	0.00 (18)	C11^i^—C10—C11—C12	179.69 (13)
N1—C1—C6—C5	−179.93 (11)	C9—C10—C11—C12	−0.31 (13)
C2—C1—C6—C5	1.96 (18)	C10—C11—C12—C13	−0.74 (18)
C5—C4—C7—O2	−175.91 (10)	C14—O1—C13—C8	−179.48 (11)
C3—C4—C7—O2	3.40 (17)	C14—O1—C13—C12	−0.25 (18)
C5—C4—C7—C8	1.21 (16)	C9—C8—C13—O1	179.77 (9)
C3—C4—C7—C8	−179.48 (10)	C7—C8—C13—O1	4.22 (15)
O2—C7—C8—C13	89.52 (13)	C9—C8—C13—C12	0.53 (17)
C4—C7—C8—C13	−87.74 (13)	C7—C8—C13—C12	−175.03 (11)
O2—C7—C8—C9	−85.85 (13)	C11—C12—C13—O1	−178.55 (11)
C4—C7—C8—C9	96.90 (12)	C11—C12—C13—C8	0.64 (19)
C13—C8—C9—C8^i^	178.46 (11)		

Symmetry codes: (i) −*x*+1, *y*, −*z*+3/2.

### Hydrogen-bond geometry (Å, °)

**Table:**

*Cg* is the centroid of the C8–C13 ring.

**Table:**

*D*—H···*A*	*D*—H	H···*A*	*D*···*A*	*D*—H···*A*
N1—H4···O1^ii^	0.93 (2)	2.26 (2)	3.1708 (17)	169.1 (18)
C14—H14B···O2^iii^	0.96	2.57	3.5013 (18)	165
N1—H3···Cg^iv^	0.92 (2)	2.50 (2)	3.3301 (13)	149.8 (18)

Symmetry codes: (ii) −*x*+3/2, −*y*+1/2, −*z*+2; (iii) −*x*+3/2, *y*−1/2, −*z*+3/2; (iv) *x*, −*y*, *z*+1/2.
